# Cardiac MRI Diagnosis of Large Aortopulmonary Collateral and Pulmonary Sequestration Presenting as Left Chamber Dilation

**DOI:** 10.1016/j.cjcpc.2025.08.002

**Published:** 2025-08-20

**Authors:** Paul W. Warren, Bradley B. Keller, Aki A. Tanimoto, Russel Hirsch, David G. Lehenbauer, Sean M. Lang

**Affiliations:** a The Heart Institute, Cincinnati Children’s Hospital Medical Center, Cincinnati, Ohio, USA; b Department of Pediatrics, University of Cincinnati College of Medicine, Cincinnati, Ohio, USA; c Division of Radiology, Cincinnati Children's Hospital Medical Center, Cincinnati, Ohio, USA; d Department of Radiology, University of Cincinnati College of Medicine, Cincinnati, Ohio, USA; e Department of Surgery, University of Cincinnati College of Medicine, Cincinnati, Ohio, USA

**Keywords:** bronchopulmonary sequestration, echocardiography, magnetic resonance imaging, vascular malformations

**Although an echocardiogram is usually sufficient to determine the etiology of left ventricular and aortic dilation in pediatric patients, cross-sectional imaging may complement echocardiography when the diagnosis is unclear. We present the case of a 2-year-old child with left atrial, left ventricular, and aortic dilation caused by an intralobar pulmonary sequestration with a large distal aortopulmonary collateral, requiring cardiac magnetic resonance imaging for diagnosis. Cardiac magnetic resonance imaging has the unique ability to provide cross-sectional anatomic information, in addition to volumetric and flow data, making it a valuable modality for the diagnostic evaluation of idiopathic chamber or vessel dilation.**

## Case Report

A 2-year-old child who recently immigrated to the United States from Honduras was referred to cardiology for a murmur evaluation. Parents denied any cardiac or respiratory symptoms, hemoptysis, or recurrent respiratory illnesses. His physical examination was notable for weight at the first percentile, a systolic click and systolic ejection murmur at the left midsternal border, normal pulses, and moderate joint hypermobility. An echocardiogram was performed, which revealed a bicuspid aortic valve with fusion of the right and left coronary leaflets, mild aortic stenosis (peak gradient 35 mm Hg and mean gradient 14 mm Hg), trivial aortic insufficiency, severe left ventricular dilation (end diastolic dimension 4.1 cm, Pediatric Heart Network *z* score +5.4), and a severely dilated aortic root (2 cm, *z* score +6.2) and ascending aorta (2 cm, *z* score +6.2). There was no difference in pulmonary vein color, spectral Doppler flow patterns, or proximal branch pulmonary artery size. The left ventricular (LV) systolic function was normal (ejection fraction 68%). At follow-up 6 months later, the patient remained asymptomatic with continued severe enlargement of the LV and aortic root.

The differential diagnosis included a left to right shunt distal to the atrioventricular valves, connective tissue disease, and inherited aortopathies.

**Figure 1:**
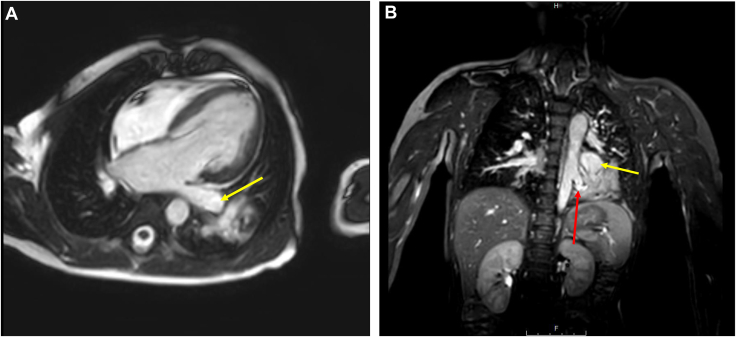
(**A**) Steady-state free precession, horizontal long-axis cine demonstrating severe left atrial and left ventricular dilation with dilation of the left lower pulmonary vein (**yellow arrow**). (**B**) Cardiac coronal cs3D BTFE whole heart sequence demonstrated a large aortopulmonary collateral (**red arrow**) arising from the left lateral aspect of the descending aorta with supply to the left lower lobe. The left lower lobe pulmonary sequestration with mass-like consolidation is also noted. There is a severely dilated and tortuous left lower pulmonary vein (**yellow arrow**). cs3D BTFE, cardiac synchronized three-dimensional balanced turbo field echo.

**Figure 2:**
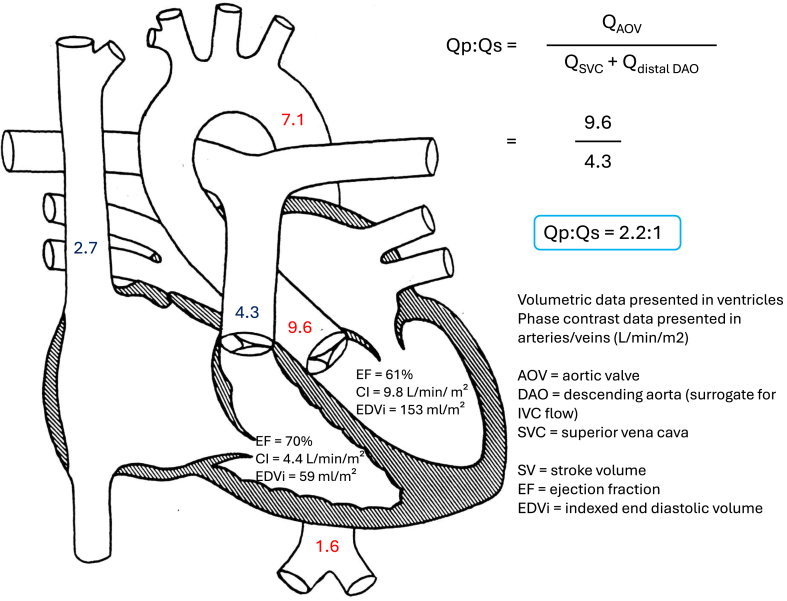
Diagram demonstrating cardiac magnetic resonance imaging volumetric and flow data. There is severe left ventricular dilation with normal systolic function. Volumetric and flow data demonstrate a 2.2:1 left to right shunt distal to the proximal descending aorta. CI, cardiac index; IVC, inferior vena cava.

The decision was made to perform cardiac magnetic resonance imaging (MRI) (1.5-Tesla magnet; Phillips Healthcare, Andover, MA) under general anesthesia to better define ventricular volumes and aortic dimensions, and investigate causes of left-sided chamber dilation. Steady-state free precession imaging confirmed severe left atrial, LV, and ascending aorta dilation in the presence of just mild aortic stenosis and trivial insufficiency (Fig. 1A). No other intracardiac abnormalities were appreciated. Phase contrast sequences were performed assessing flows at the aortic valve, main pulmonary artery, superior vena cava, ascending aorta and descending aorta proximally, and at the level of the diaphragm. Volumetric and flow data were used to calculate a Qp:Qs (ratio of pulmonary flow to systemic flow) of approximately 2.2:1, with shunting distal to the semilunar valves and at the proximal descending aorta (Fig. 2). Dynamic as well as postcontrast 3-dimensional respiratory-navigated and electrocardiogram-gated angiography demonstrated a large left lower lobe pulmonary sequestration with a large aortopulmonary (AP) collateral feeding the left lower lobe. There was associated left lower pulmonary vein tortuosity and severe dilation (Fig. 1B).

A cardiac catheterization was performed to attempt AP collateral occlusion, but this was unsuccessful because of the size and tortuosity of the feeding vessels. The patient was thus referred for surgical intervention. The patient underwent left lower lobectomy via thoracotomy and ligation and division of the large AP collateral. Intraoperatively, the native pulmonary arteries to the left lower lobe were diminutive, and the left lower lobe parenchyma was noted to have atypical hypervascularization. Surgical pathology confirmed an intralobar pulmonary sequestration. He had an uncomplicated convalescence.

The echocardiogram was repeated at the last clinic follow-up 8 months after surgical repair; his ascending aorta remained severely dilated, but he had normalization of his LV dimensions and function and normalized aortic valve velocities. Serial follow-up was arranged for ongoing monitoring of his bicuspid valve and aortic dilatation.

## Discussion

We present the case of a 2-year-old child with left-sided chamber and aortic dilation that is out of proportion to and inconsistent with his degree of aortic valve disease. Cardiac MRI aided in the eventual diagnosis of pulmonary sequestration and a hemodynamically significant distal AP collateral.

Pulmonary sequestration is a rare pulmonary and vascular malformation, accounting for 1%-8% of all congenital lung malformations.^1^ It is defined as pulmonary parenchyma that is nonfunctioning and supplied by a systemic artery.^2^ Clinical presentation varies, with many patients asymptomatic, while others present with hemoptysis or congestive heart failure due to left-to-right shunting.^3^ Management includes surgical lobectomy or catheter-based occlusion of the arterial feeder vessel.^4^ Interventional mortality is low.^3^

This case highlights the utility of cardiac MRI in determining the precise etiology of chamber enlargement that is not explained by echocardiogram findings. The lack of significant aortic insufficiency and mild degree of aortic stenosis could not account for the severity of LV and aortic dilation in our patient. In most cases of pulmonary sequestration, the aberrant artery feeding the abnormal lung mass arises from the descending aorta, making diagnosis by echocardiography alone challenging.^5^ Cardiac MRI allowed us to estimate the Qp:Qs, which revealed evidence of a significant left-to-right shunt and ultimately led to the diagnosis. Importantly, cardiac MRI provided these data without the need for an invasive diagnostic procedure.

In conclusion, cross-sectional imaging is useful for the diagnostic evaluation of children with left-sided chamber and aortic dilation that is out of proportion to echocardiogram findings. This case highlights the ability of cardiac MRI to provide anatomic, volumetric, and flow data without the need for invasive diagnostic procedures.

**Novel Teaching Points**

• The differential diagnosis for left-sided chamber and aortic dilation in children that is not explained by intracardiac pathology includes left to right shunts anywhere distal to the atrioventricular valves, including distal aortopulmonary collaterals.
• Cardiac MRI can complement echocardiography to facilitate accurate diagnosis of complex heart disease without the need for cardiac catheterization.
